# Modified biweekly cisplatin, docetaxel plus cetuximab (TPEx) as first-line treatment for patients with recurrent/metastatic head and neck cancer

**DOI:** 10.1007/s12032-018-1087-6

**Published:** 2018-02-07

**Authors:** Hannah Fuchs, Johannes Pammer, Christoph Minichsdorfer, Doris Posch, Gabriela Kornek, Marie-Bernadette Aretin, Thorsten Fuereder

**Affiliations:** 1 Division of Clinical Oncology, Department of Internal Medicine I & Comprehensive Cancer Center, Medical University of Vienna, Währinger Gürtel 18-20, 1090 Vienna, Austria; 2 Department of Pathology, Medical University of Vienna, Währinger Gürtel 18-20, 1090 Vienna, Austria; 3 Pharmacy Department, General Hospital Vienna, Währinger Gürtel 18-20, 1090 Vienna, Austria

**Keywords:** Recurrent and/or metastatic head and neck cancer, Palliative chemotherapy, Cetuximab, Docetaxel, Cisplatin

## Abstract

Three weekly high-dose chemotherapy regimens in combination with weekly cetuximab are the treatment of choice for patients with recurrent/metastatic (R/M) head and neck squamous cell carcinoma (SCCHN), although the majority of patients suffer from severe side effects. Thus, we investigated the efficacy and safety of an alternative, more convenient and less toxic biweekly modified cisplatin, docetaxel plus cetuximab (TPEx) regimen in this retrospective analysis. Thirty-eight patients receiving off-protocol cisplatin (50 mg/m^2^) in combination with docetaxel (50 mg/m^2^) plus cetuximab (500 mg/m^2^) every other week were included. Data collection included baseline demographic, response rate (ORR) and toxicity data as well as disease control rate, overall survival (OS) and progression-free survival (PFS). The median age was 60 years, and the majority of patients suffered from oral cavity carcinomas (44.7%) followed by oropharyngeal (28.9%) and laryngeal (17.9%) carcinomas. The ORR was 50%, and four (10.5%) patients achieved a complete response, while 15 (39.5%) patients had a partial response. The OS and PFS were 10.8 months (95% CI 6.7–14.2) and 6.3 months (95% CI 5.7–6.8), respectively. The one-year survival rate was 44.7%. The therapy was well tolerated, and the most common grade 3/4 adverse events were myelosuppression (13.2%), hypomagnesaemia (23.7%) and acne-like rash (13.1%). In conclusion, modified biweekly TPEx is of comparable efficacy with conventional TPEx and represents a well-tolerated regimen in R/M SCCHN patients. Further evaluation of this protocol in prospective clinical trials is warranted.

## Introduction

For stage III/IV squamous cell carcinoma of the head and neck (SCCHN), the locoregional recurrence rate after curative therapy was reported to be 30–40% [1]. Distant metastases at diagnosis (mainly pulmonary) are detected only in a minority of patients [2]. Additionally, second primaries occur at a constant rate of 2–3% per year [3, 4]. Attempts to implement adjuvant treatment strategies after chemoradiation in order to improve outcomes such as the epidermal growth factor receptor (EGFR) inhibitor afatinib have not been successful so far [5].

For patients, who are not amenable to salvage surgery, only limited palliative treatment options exist: globally, systemic combination therapy is the category I recommendation for patients with locoregionally unresectable recurrent or metastatic (R/M) disease and excellent performance status.

During the last decade, chemotherapy regimens have been constantly improved and novel drugs such as the EGFR antibodies panitumumab or cetuximab were evaluated in clinical trials for the treatment of R/M SCCHN patients. However, the median overall survival (OS) in this setting is still about 8–10 months employing the EXTREME regimen, which is containing a platinum drug, 5-FU and weekly cetuximab and regarded as the standard of care [6].

Apart from platinum drugs and cetuximab, taxanes were shown to exert significant anti-tumor activity against SCCHN cells. Numerous clinical trials demonstrated the efficacy of both docetaxel and paclitaxel as single agents and in combination with platinum drugs in R/M SCCHN [7].

The molecular basis of clinical reports, which showed synergistic effects of taxanes in combination with cetuximab, is not fully understood. Various molecular mechanisms such as the prevention of taxane induced EGFR phosphorylation or modulation of the EGFR downstream pathways by taxanes might contribute to the beneficial activity profile of this particular combination [8, 9]. From the clinical point of view, both docetaxel and paclitaxel in combination with cetuximab are an effective regimen for R/M SCCHN patients both in the first-line and in the second-line setting accompanied with a beneficial side-effect profile [10, 11].

A recent phase II study demonstrated that the substitution of 5-FU by docetaxel in combination with cisplatin given every 3 weeks plus weekly cetuximab might be as effective as the EXTREME regimen in R/M SCCHN patients and is well tolerated [12]. However, further streamlining and simplifying this dosing regimen would be of particular value with respect to patients’ quality of life in the palliative setting.

Based on these considerations, we performed this retrospective analysis in order to evaluate the efficacy and safety of modified cisplatin, docetaxel plus cetuximab (TPEx) administered every other week as first-line therapy in patients suffering from R/M SCCHN.

## Patients and methods

### Data collection

 Patients eligible for this single-center retrospective analysis had histologically or cytologically confirmed R/M SCCHN diagnosed between January 1, 2007, and December 31, 2016, at the Medical University of Vienna. Prior chemotherapy for advanced disease, other than squamous histology and sites other than laryngeal, hypopharynx, oropharynx and oral cavity were exclusion criteria. Previous taxane therapy as part of induction chemotherapy before radiotherapy was allowed.

Demographic and clinical data including patients’ age, ECOG performance status, clinical stage, tumor response, chemotherapy cycles administered, survival data and toxicity data were collected retrospectively from patients’ notes and prescription charts. The study was performed in accordance with the Declaration of Helsinki and good clinical practice guidelines and was approved by the Ethics Committee of the Medical University of Vienna (#1679/2017).

### Treatment protocol

Chemotherapy consisted of biweekly docetaxel 50 mg/m^2^ diluted in 250 ml saline administered as a 60-min intravenous infusion, cisplatin 50 mg/m^2^ diluted in 1000 ml saline administered as a 120-min intravenous infusion plus cetuximab 500 mg/m^2^ administered as a 120-min intravenous infusion on day one. Chemotherapeutic/cetuximab treatment courses were repeated every 2 weeks until disease progression, unacceptable toxicity or patient’s request for treatment discontinuation. Ondansetron, dexamethasone, aprepitant and diphenhydramine were routinely given as premedication.

Radiographic imaging employing computed tomography or magnetic resonance imaging was performed at baseline and at 12-week intervals until disease progression. Treatment response was evaluated according to RECIST 1.1 criteria by an independent radiologist. Adverse events were graded according to the National Cancer Institute Common Terminology Criteria for Adverse Events (version 4.0).

### Statistical analysis

Statistical analysis was performed employing SPSS 23 software package (SPSS Inc., Chicago, IL, USA). Continuous variables were shown using descriptive statistics. Categorical variables were summarized using percentages and counts. For survival analysis, including PFS and OS, the Kaplan–Meier method was used for univariate analysis. The data for patients who were alive were censored at the time of last confirmed contact.

## Results

### Patient characteristics

In this retrospective study, 38 patients with recurrent/metastatic squamous cell carcinoma of the head and neck not amenable for curative treatment were analyzed. All patients received off-protocol first-line chemotherapy with cisplatin, docetaxel and cetuximab given every other week between 2007 and 2016 at the Medical University of Vienna. Table 1 depicts demographic data, baseline disease characteristics, prior curative treatment including surgical procedures and radiotherapy. Our patient cohort represents a typical SCCHN population. Patients were predominantly male (82%) and heavy smokers (i.e., over 10 pack years). While 26 patients (81.3%) were current of former smokers, 16 patients (50%) had a history of alcohol abuse in their medical records. This was an elderly population with a median age of 60 years (range 42–74 years). All patients had an ECOG performance status of 0 or 1.

**Table 1:** Patient and disease characteristics at baseline

Characteristics	Number of Patients (%)
Sex	
Male	31 (82%)
Female	7 (18%)
Median age (range)	60 years (42–74)
Nicotine abuse	
Yes	26 (81.3%)
No	6 (18.7%)
Not evaluable	6
Alcohol abuse	
Yes	16 (50%)
No	16 (50%)
Not evaluable	6
Primary tumor site	
Hypopharynx	1 (2.6%)
Oral cavity	17 (44.7%)
Oropharynx	11 (28.9%)
Larynx	5 (17.9%)
Double locations	4 (10.5%)
p16 status (oropharyngeal carcinoma)	
Positive	5 (45.5%)
Negative	6 (54.5%)
Cycles (range)	3 (1–7)
Median duration of treatment	2.6 months
Previous treatment	
Primary treatment	
No primary treatment	4 (10.5%)
Surgery alone	8 (21.1%)
Surgery plus radiotherapy	16 (42.1%)
Surgery plus concomitant chemoradiotherapy	1 (2.6%)
Concomitant chemoradiotherapy	5 (13.2%)
Radioimmunotherapy	1 (2.6%)
Neoadjuvant chemotherapy plus surgery	1 (2.6%)
Ind. chemotherapy plus radioimmunotherapy	1 (2.6%)
Ind. radioimmunotherapy plus surgery (Study)	1 (2.6%)
Salvage treatment of recurrence	
All	13
Surgery	6 (46.2%)
Surgery plus radiotherapy	6 (46.2%)
Radioimmunotherapy	1 (7.7%)
Extent of disease	
Locoregional recurrence alone	14 (36.8%)
Metastatic disease alone	6 (15.8%)
Locoregional recurrence + metastatic disease	18 (47.4%)

The majority of the patients suffered from oral cavity carcinomas (44.7%) followed by oropharyngeal (28.9%) and laryngeal (17.9%) carcinomas. In patients with oropharyngeal carcinoma, p16 was detected in five cases. Of note, while only one (2.6%) patient was diagnosed with hypopharyngeal cancer, tumor recurrence at multiple sites was observed in four (10.5%) patients. Synchronous metastases were detected in 18 (47.7%) patients with locoregional recurrence, whereas 14 (36.8%) patients suffered from locoregional failure only. Distant metastases (primarily pulmonary metastases) without evidence of recurrence at the primary tumor sites were observed in six (15.8%) patients. The majority of patients (42.1%) received surgery plus adjuvant radiotherapy or surgery alone (21.1%) as the initial curative treatment strategy. Upon relapse, one or more salvage treatments were performed in 13 (34%) patients prior to systemic chemotherapy.

The median number of chemotherapy cycles with cisplatin, docetaxel and cetuximab was three with the range one to seven.

### Tumor response and survival

Three months after treatment initiation, a restaging CT scan or MRI was performed in order to assess objective response.

We detected four (10.5%) CRs, 15 (39.5%) partial responses and two (3.5%) stable diseases. Therefore, an ORR of 50% was achieved. The majority of the patients benefited from this regimen, since the DCR was 53.5%. Only a minority of the patients (18.4%) experienced disease progression. However, 10 (26%) patients were not evaluable for the analysis due to early death prior to imaging (8) most probably because of rapid tumor progression or lost to follow-up (2) (Table 2).

**Table 2:** Summary of treatment results

Best response	Number of patients (%)
CR	4 (10.5%)
PR	15 (39.5%)
SD	2 (3.5%)
PD	7 (18.4%)
Not evaluable	10 (26%)
Death prior to imaging	8
Lost to follow-up	2

**Fig. 1:**
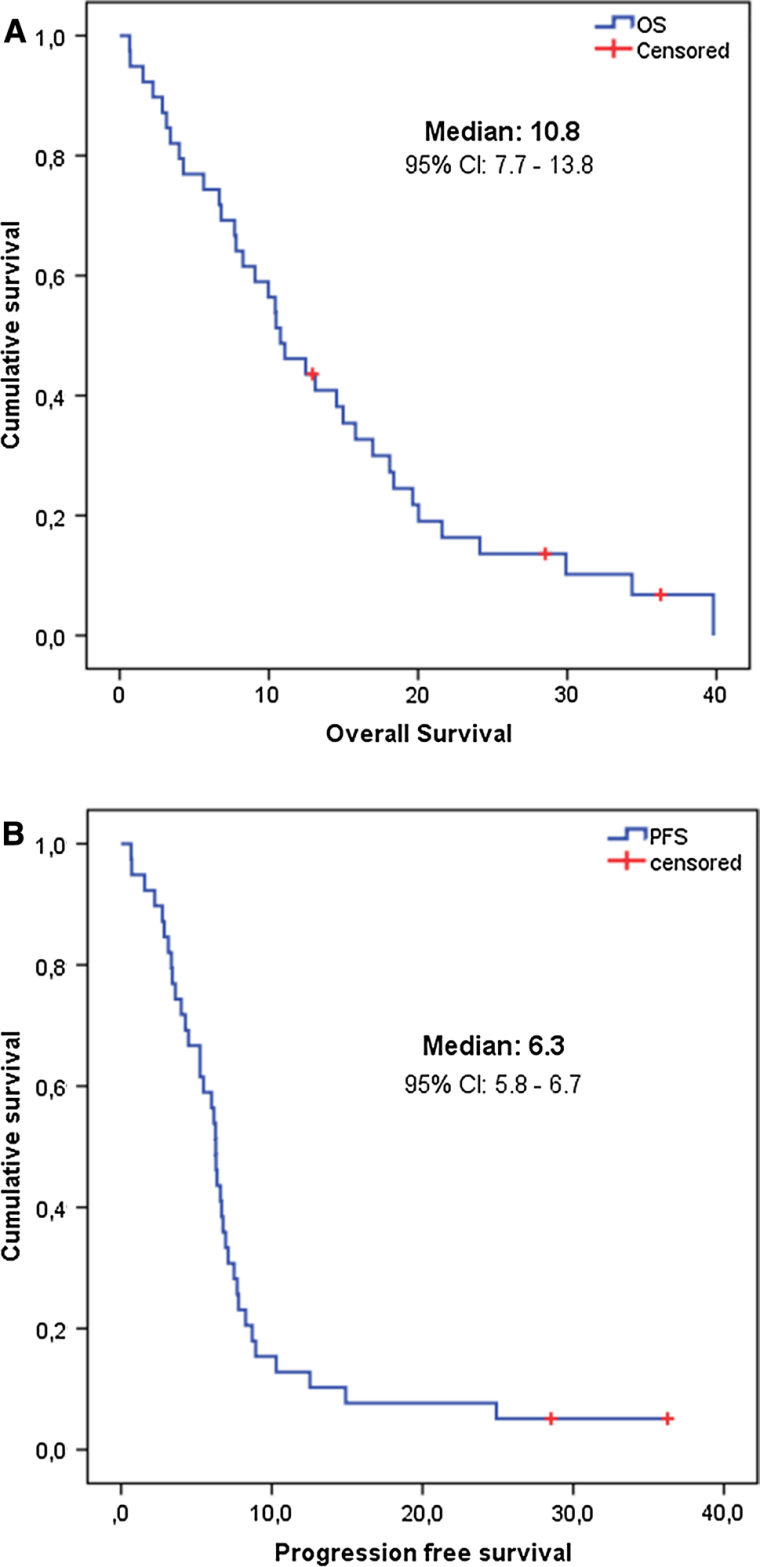
Kaplan–Meier curves depicting overall survival (**a**) and progression-free (**b**) survival

The median overall survival was 10.8 months (95% CI: 6.7–14.2 months), and the median progression-free survival was 6.3 months (95% CI: 5.7–6.8 months) (Fig. 1a, b). The one-year survival rate was 44.7%.

### Safety and tolerability

Overall biweekly cisplatin, docetaxel plus cetuximab was well tolerated and no new safety issues arose. Grade 3–4 adverse events, which were treatment-related, occurred in 38 cases (Table 3). Hypomagnesemia (23.7%), hypokalemia (13.1%), neutropenia (13.1%) and anemia (10.5%) were the most common ones. Two patients developed an allergic reaction to cetuximab, five patients had acne-like rush, and three patients suffered from bacterial infections. Gastrointestinal toxicities such as diarrhea were reported in three cases.

**Table 3:** Grade 3 to 4 treatment-related adverse events

Adverse event	Number of patients (%)
All	38
Anemia	4 (10.5%)
Neutropenia	5 (13.2%)
Hypomagnesemia	9 (23.7%)
Hypokalemia	5 (13.1%)
Acne-like rash	5 (13.1%)
Allergic reaction	2 (5.3%)
Diarrhea	2 (5.3%)
Nausea	3 (7.9%)
Pneumonia	2 (5.3%)
MRSA plus fever	1 (2.6%)

## Discussion

Despite recent advances in immunotherapy and extensive research efforts, R/M head and neck cancer is still a clinical challenge. Since no curative treatment options are available in this setting, prolonging OS and symptom control is the ultimate treatment goal. In this retrospective analysis, we show that a modified TPEx regimen administered biweekly is a feasible, safe and effective regimen in unselected patients suffering from R/M SCCHN.

In the first-line platinum-sensitive setting, a combination chemotherapy according to the EXTREME regimen is regarded as the standard of care worldwide, although considerable shortcomings are linked to this protocol such as the high amount of patients (82%) suffering from severe grade 3/4 adverse events or the continuous administration of 5-FU for four days [6]. Two recently published phase II studies demonstrated that substitution of 5-FU by a taxane might be equi-effective with respect to OS but yield in higher ORR and better toxicity profile [12, 13]. The first study enrolled 54 patients and reported that conventional TPEx consisting of cisplatin/docetaxel given every 3 weeks plus cetuximab administered weekly results in an OS of 14 months (95% CI 11.3–17.3) and an ORR of 44% [12]. However, two infectious events leading to death were observed [12]. The second study compared three weekly cisplatin plus weekly cetuximab with or without paclitaxel resulting in a median OS of 11 month, a median PFS of 7 months and an ORR of 51.7% in the cisplatin/paclitaxel plus cetuximab group [13]. Given the limitations of inter-trial comparisons, the low patient number and the retrospective nature of our analysis these results are completely in line with our findings. Patients receiving off-protocol modified biweekly TPEx had a median OS of 10.8 months (95% CI 6.7–14.2) and a median PFS of 6.3 months (95% CI 5.7–6.8) indicating a comparable efficacy of the biweekly TPEx regimen. Tumor shrinkage is a crucial issue for head and neck cancer patients leading to symptom relief and quality of life improvement, although response rates do not always translate into a prolonged OS as shown in the aforementioned trial. While the ORR of biweekly TPEx was 50% in our analysis, conventional TPEx was reported to yield in an ORR of 44.4%. ORR with cisplatin in combination with paclitaxel and cetuximab was numerically higher (51.7%), whereas the standard of care EXTREME regimen achieved tumor response in 36% of the patients [6]. Of note, we observed four complete responders, whereas in the conventional TPEx regimen, no CRs were reported [12]. This finding further supports the activity of modified TPEx given every other week. Interestingly, the DCR of 53.5%, we observed in our analysis was inferior compared to the other two cisplatin/taxane/cetuximab trials, which reported a DCR of 79.6 and of 76.4%, respectively [12, 13]. Additionally, compared to the conventional TPEx study, a higher amount of patients were not assessable for response due to early death (26 vs. 11%) in our analysis. We are aware that making assumptions for the definitive reasons for this discrepancy is speculative and no final conclusions can be drawn given the retrospective nature of this analysis. However, we have to point out that a higher fraction of our patients received salvage treatment for recurrence (34%) and/or upfront multimodality treatment such as surgery plus radiotherapy or chemoradiation (44%), thus representing a patient population at increased risk for rapid disease progression.

Modified biweekly TPEx was very well tolerated and no new safety issues arose. Although the majority of patients suffered from a grade 3/4 adverse event, the side effects were well manageable. As compared to the conventional TPEx study, we observed lower rates of severe neutropenia (13%), which is in line with other trials employing a biweekly platinum/taxane protocol [14].

However, the main advantage of biweekly TPEx should be considered both under quality of life and health economic aspects: Minimizing the number of outpatient visits will reduce health care costs and the need for health care staff and is more convenient for the patient than weekly infusions. From the pharmacological point of view, several trials have shown that biweekly schedules are feasible and might be equivalent to weekly or three weekly regimens in the palliative setting: For cetuximab, it was shown that there is no difference between biweekly and weekly cetuximab in target regulation, pharmacokinetics and pharmacodynamic parameters in colorectal cancer and head and neck cancer patients [15, 16]. Likewise, it has been demonstrated that cisplatin/docetaxel biweekly is as effective as cisplatin/docetaxel triweekly accompanied with fewer side effects in non-small cell lung cancer patients [14].

In conclusion, modified biweekly TPEx is a feasible and effective regimen for the first-line therapy of R/M SCCHN patients. Non-inferiority trials are warranted in order to establish this regimen as a standard of care treatment protocol for R/M SCCHN.
